# Respiratory, birth and health economic measures for use with Indigenous Australian infants in a research trial: a modified Delphi with an Indigenous panel

**DOI:** 10.1186/s12887-020-02255-x

**Published:** 2020-08-05

**Authors:** Sarah Perkes, Billie Bonevski, Joerg Mattes, Kerry Hall, Gillian S. Gould

**Affiliations:** 1 Hunter Medical Research Institute and School of Medicine and Public Health, Faculty of Health and Medicine, University of Newcastle, University Drive, Callaghan, New South Wales 2308 Australia; 2 First Peoples Health Unit, (FPHU) Griffith University, Southport, Queensland 4215 Australia

**Keywords:** Indigenous, Infant, Respiratory, Measures

## Abstract

**Background:**

There is significant disparity between the respiratory health of Indigenous and non-Indigenous Australian infants. There is no culturally accepted measure to collect respiratory health outcomes in Indigenous infants. The aim of this study was to gain end user and expert consensus on the most relevant and acceptable respiratory and birth measures for Indigenous infants at birth, between birth and 6 months, and at 6 months of age follow-up for use in a research trial.

**Methods:**

A three round modified Delphi process was conducted from February 2018 to April 2019. Eight Indigenous panel members, and 18 Indigenous women participated. Items reached consensus if 7/8 (≥80%) panel members indicated the item was ‘very essential’. Qualitative responses by Indigenous women and the panel were used to modify the 6 months of age surveys.

**Results:**

In total, 15 items for birth, 48 items from 1 to 6 months, and five potential questionnaires for use at 6 months of age were considered. Of those, 15 measures for birth were accepted, i.e., gestational age, birth weight, Neonatal Intensive Care Unit (NICU) admissions, length, head circumference, sex, Apgar score, substance use, cord blood gas values, labour, birth type, health of the mother, number people living in the home, education of mother and place of residence. Seventeen measures from 1-to 6 months of age were accepted, i.e., acute respiratory symptoms (7), general health items (2), health care utilisation (6), exposure to tobacco smoke (1), and breastfeeding status (1). Three questionnaires for use at 6 months of age were accepted, i.e., a shortened 33-item respiratory questionnaire, a clinical history survey and a developmental questionnaire.

**Conclusions:**

In a modified Delphi process with an Indigenous panel, measures and items were proposed for use to assess respiratory, birth and health economic outcomes in Indigenous Australian infants between birth and 6 months of age. This initial step can be used to develop a set of relevant and acceptable measures to report respiratory illness and birth outcomes in community based Indigenous infants.

## Background

Indigenous Australian children experience unacceptably high rates of respiratory disease [1–4]. Up to 1 in 3 Indigenous infants are hospitalised for acute respiratory infections in their first year of life [5]. Rates of chronic respiratory disease are also high among Indigenous children, including asthma (19.5%), bronchitis (16.8%), bronchiolitis (12.2%), pneumonia (7.2%) and bronchiectasis (1.5%) [6]. Poor respiratory health continues across the lifetime for Indigenous Australians leading to a shorter and poorer quality of life. In 2011–15 there were 1092 respiratory disease deaths among Indigenous Australians (8% of Indigenous deaths), twice the non-Indigenous rate [7].

A combination of social, historical, and cultural contexts contribute to the high, and unacceptable rates of disease [2]. Risk factors include overcrowding, malnutrition, young maternal age, low birthweight, anaemia, poverty, illiteracy, overcrowding, exposure to tobacco smoke and parental smoking [8], pollution, socioeconomic status, social behaviours, cultural exposure, family history, and a history of prior illness [2]. Addressing the social determinants of health will see the greatest reduction in respiratory disease among Indigenous children, though clinical care must be improved simultaneously [2, 9].

Despite respiratory disease being a leading contributor to the total burden of disease among Indigenous children, there is scarcity of community level data [2]. One single urban centre study with 180 Indigenous children under 5 y of age used monthly interviews over 12 months to measure acute respiratory illness [10]. One in five children experienced at least one episode of chronic cough [11]. More than half of the children identified with chronic cough were diagnosed with an underlying lung disease, mostly protracted bacterial bronchitis, asthma and bronchiectasis [11]. A second study in remote Indigenous communities with 651 children under 6 y of age using observations to measure illness reported a point prevalence for cough (acute or chronic) of 39% [3]. In national parent reported data from 2012 to 2013 asthma prevalence is 15% as compared to 9% in non-Indigenous children [12].

As well as limited data, inconsistent measures have been used to capture respiratory illness. There are no standard measures for respiratory symptoms or illness specifically developed for Indigenous children [2]. In research trials, respiratory symptoms are typically collected via parent-reported questionnaires, interviews, or symptom diary cards [13]. Parent-reported measures are valuable and clinically relevant with wide reach at relatively low cost. However parent-report is reliant on accurate recall and health literacy and response rates can be low [14]. Cough is the main outcome collected via parent-report for respiratory illness [13]. Reliability of parent reported cough for children is reported to be good for daytime cough and poor for nocturnal cough [13]. Accuracy of parent reported wheeze is reported to be low [15]. Gold standard measures for detecting respiratory illness are clinical assessment including observation and objective tests such as spirometry and/or x-ray [16], though these measures can be impractical for trials due to the ongoing and fluctuating nature of symptoms as well as being costly, time intensive and burdensome for families.

Culturally safe, effective measures for detecting respiratory illness in Indigenous infants needs further development to improve respiratory health outcomes [2]. Accurate data is vital to enable us to understand the current state of Indigenous infant health, to acknowledge progress, and to determine how to reduce inequalities between Indigenous and non-Indigenous children [17]. There is an entrenched lack of trust from Indigenous Australians in health care professionals and systems [18], medical research [19] due to historical and current policies (including the Stolen Generations) which requires intense consultation with Indigenous leaders, consumers and topic experts to ensure that cultural safety of Indigenous peoples is paramount in research [19]. The purpose of this study was to systematically consult a group of Indigenous academics, clinicians and women on the most accurate, culturally safe, and feasible respiratory health measures for use with Indigenous mothers and infants for a research trial.

## Method

### Study design

A modified Delphi with an Indigenous expert panel was used. The Delphi method is a culturally acceptable method of gaining consensus and has been used in other areas of Indigenous health research [20, 21]. The consensus process was completed between February 2018 and April 2019. The Delphi technique is a method used to collect opinions from a group of experts to achieve consensus on a particular research question [22]. Repeated questionnaires are used to facilitate independent, gradual and considered opinions [23]. Modified versions involving group discussion may be used where feasibility and operational aspects are solved through group problem solving [24–26]. In this study, discussion was also an opportunity for dialogue on cultural safety considerations. This study was conducted in the context of identifying Australian Indigenous culturally acceptable measures for use in a trial to assess infant respiratory symptoms and illness. The measures would be used to follow up infants born to mothers enrolled in the SISTAQUIT® (Supporting Indigenous Smokers To Assist Quitting) smoking cessation trial (Australian New Zealand Clinical Trials Registry trials (ACTRN12618000972224).

### Participants

An Indigenous expert panel participated in the three round Delphi process and Indigenous women provided feedback on the 6-month surveys. Using a snowball recruitment strategy, a list of 12 potential expert panel members known to study Investigators were invited to participate by email. The 12 potential participants were sent a summary of the study and asked to share the invitation with colleagues. Twenty Indigenous health organisations were also contacted via email and phone and invited to participate. Eight panel members agreed to participate in total. The 8 panel members were emailed the full SISTAQUIT study protocol prior to the first round. Panel members included, 1) Postdoctoral researcher in acute respiratory illness with Indigenous children, 2) Principal Research Fellow in mothers and babies health, 3) representative of HealthInfoNet, 4) Associate Professor at an Indigenous research unit 5) Representative of Indigenous Allied Health Australia (IAHA), 6) Obstetrician, 7) Paediatrician and 8) Representative of The Congress of Aboriginal and Torres Strait Islander Nurses and Midwives (CATSINaM). The 8 panel members participated in the each round for each measurement tool, with the exception of 1 participant who did not attend round 1.

Indigenous women (*n* = 18) were recruited as part of a separate study (unpublished) on resources used for Indigenous women’s and child’s health. Women were recruited through known networks of Indigenous research assistants in Hunter New England and the Mid North Coast of New South Wales. Women were 16 years or over and mothers of young children.

### Description of the modified Delphi method used

**Fig. 1:**
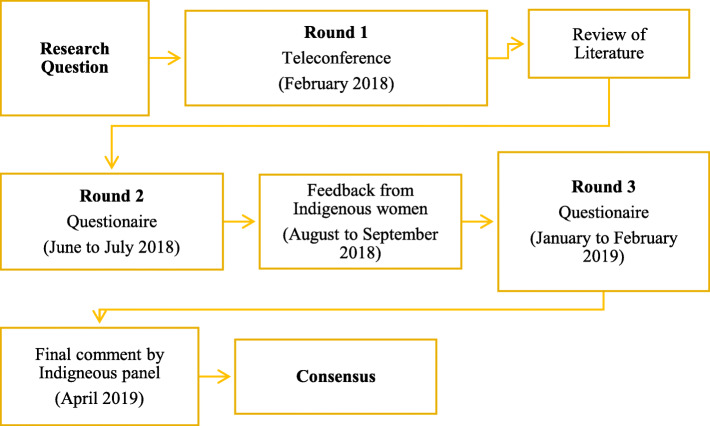
Overview of consensus process

A three round modified Delphi with teleconference and two repeat questionnaires was used. An overview of the consensus process is presented in Fig. 1. Round one involved a group discussion with the Indigenous expert panel and rounds two and three used repeat online questionnaires. Feedback from 18 Indigenous women on potential respiratory questionnaires for use at 6 months of age were gathered between rounds two and three.

### Review of literature

The lead author (SP) reviewed the literature to identify outcome measures used with Indigenous Australian infants up to 6-months of age. Outcomes of interest were 1) birth outcomes related to adverse impact of exposure to tobacco in-utero (as per broader study), 2) respiratory symptoms and illness, 3) health care utilisation, and 4) developmental outcomes. Keywords were used to search electronic databases including HealthInfoNet, Google Scholar, ScienceDirect, Cochrane Library and CINAHL. Reference lists and grey literature were searched. Known experts in the field were contacted and asked of knowledge on measures used in clinical practice.

### Round one: teleconference

The first teleconference was used to provide an overview of the study; and to seek preferences for the Delphi process i.e. online questionnaires or interviews. During this call, participants were also asked to share knowledge on potential measures and were given guidance on the information required by the panel to support decision-making.

### Questionnaire development

The questionnaire of potential outcomes included items on types of outcome measures, mode and frequency of data collection and acceptability of existing surveys for use at 6-months of age. Potential birth outcome measures were derived from a Cochrane review on smoking cessation interventions used during pregnancy [27], acute respiratory symptoms from a survey used in a longitudinal study on respiratory symptoms in Indigenous children [10] and items on health care utilisation from a systematic review and a cost-consequence analysis [28, 29]. Two additional items on breastfeeding and exposure to environmental tobacco smoke were added from the respiratory symptoms survey [10]. Potential questionnaires identified for use from a literature review at 6 months included two respiratory screening tools, 1) a 50-item respiratory questionnaire [30] and 2) an 18-item respiratory adapted into Creole [31] as well as a clinical assessment form developed for the purpose of the larger study. A development screening tool with an adapted version for remote Indigenous communities was also identified [32, 33]. A Respiratory Paediatrician (JM) and Health Research Economist (SD) provided expertise on respiratory health and health care utilisation items respectively.

### Round two: questionnaire

An online questionnaire delivered on REDCap software was used. The questionnaire consisted of three sections with 58 items. Participants were also asked for feedback on four existing questionnaires for use at 6 months of age. In total, participants took approximately 30 min to complete. In section one, participants were asked to answer two multiple-choice items. The first to identify measures to be collected at birth including birth weight, gestational age, Apgar score, Neonatal Intensive Care Unit (NICU) admissions, sex, length, and head circumference. The second item to identify how to collect birth information including hospital discharge summary or data linkage. Consensus was pre-determined for multiple-choice items as 80% agreement [25, 34]. Items were included if 80% agreement was reached (7/8 participants selected a measure), items progressed to round three if agreement was between 50 and 80% (4 to 6 participants selected a measure) and omitted if below 50% (less than four participants selected a measure). Two open-ended questions were also included in section one on additional measures to collect at birth and other modes of data collection. Additional items suggested in qualitative responses were added to the round three questionnaire.

In section two, participants were asked to rate respiratory symptoms and health care utilisation items using a 4-point Likert scale (very essential, somewhat essential, non-essential and unsure) as to whether each item should be collected in the trial. As above, consensus was pre-determined as 80% agreement (using ‘very essential’ only). Items progressed to round three if agreement was 50 to 80% and omitted if below 50%. In the final section, participants were asked for qualitative feedback on 4 potential questionnaires for use at 6-months: two respiratory, one developmental and one clinical assessment form. Qualitative responses were synthesised and used to modify questionnaires.

### Feedback from indigenous women

Two focus groups were held by Indigenous research assistants to gain feedback from 18 Indigenous women on two respiratory questionnaires. Both focus groups were conducted in regional areas of New South Wales. The focus groups were part of a separate study on resources used for Indigenous women’s and child’s health Women were 16 years or older, and were all mothers of young children. Questions used to gather feedback on acceptability include: 1) Are the questions easy to understand? 2) Is the language appropriate? 3) What do you think of the length of the questionnaire? 4) Would you feel comfortable answering this questionnaire? Women provided feedback verbally and in writing. Feedback was used to modify questionnaires.

### Round three: questionnaire

The round three survey was sent via email to the panel and took participants approximately 45 min to complete. Additional information was provided as requested by participants in round two to aid decision making. In section one, participants were asked to indicate ‘yes’ or ‘no’ for inclusion of additional birth measures added by participants in round two (substance use in pregnancy, cord blood gas values, labour (induction, spontaneous), birth type (caesarean, vaginal), health of mother, number people living in home, education and place of residence). Items were included if 80% agreement was reached (7/8 participants selected a measure) and omitted if below 50% (less than four participants selected a measure). If consensus was not reached a fourth round would have been conducted over phone or email.

In section two, participants were asked to rate respiratory symptoms and health care utilisation items that had not reached consensus in round two using a 4-point Likert scale. A rule was enacted to combine ‘very essential’ and ‘somewhat essential’ responses. Items that reached 80% agreement when ‘very essential’ and somewhat essential’ were combined were included. This rule was not pre-determined and enacted due to the timeline of the larger study.

In section three, participants were provided summary points of the qualitative feedback as well as the modified versions of the three questionnaires and asked to indicate ‘yes’ or ‘no’ for the acceptability of the modified versions. A space was available for qualitative feedback. The final questionnaires were presented to the panel. If consensus had not been reached a fourth round would have been conducted over phone or email.

## Results

### Round one: teleconference

Four of the eight panel members attended a group teleconference and three members were interviewed individually by SP. The panel agreed to participating in online questionnaires rather than interviews to increase flexibility in participation for future rounds. The panel recommended qualitative feedback be included as well as the rating of items.

#### Birth outcomes

### Round one: teleconference

Birth outcomes discussed as important included birth weight, small for gestational age, head circumference Apgar score, delivery at less than 37 weeks gestation, stillbirth, NICU admissions and sex. Panel members considered it essential to limit women’s burden to answer surveys straight after birth by using discharge summaries or data linkage.

### Round two: questionnaire

Seven measures at birth (birth weight, gestational age, Apgar score, NICU admissions, sex, length, head circumference) were presented for consensus. Three items reached consensus and four progressed to round three (Table 1). The panel suggested an additional seven outcomes in qualitative responses including substance use in pregnancy, cord blood gas values, labor type (induction, spontaneous), birth type (caesarean, vaginal), health of the mother, number people living in home, educational attainments of the mother and place of residence. Seven members (> 80%) indicated the best mode of data collection to be hospital discharge summary.

**Table 1:** Consensus for birth outcomes

Items	Round 2 ***n*** = 8	Round 3 ***n*** = 8	Consensus
Gestational age	7	–	✓
Birth weight	7	–	✓
NICU admissions	7	–	✓
Length	6	7	✓
Head circumference	4	8	✓
Sex	5	8	✓
Apgar score	5	8	✓
Substance use in pregnancy	–	8	✓
Cord blood gas values	–	8	✓
Labour (induction, spontaneous)	–	8	✓
Birth type (caesarean, vaginal)	–	8	✓
Health of mother	–	8	✓
Number people living in home	–	8	✓
Education	–	8	✓
Place of residence	–	8	✓
**Total:**	**7**	**12**	**15**

Data collection from one to 6 months of age for respiratory symptoms and health service utilisation

### Round three: questionnaire

Twelve items were presented for consensus (Table 1). All 12 items in round 3 reached consensus (Table 1). A total of 15 items were accepted as essential items to collect. (see Additional file 1 for data extraction form).

### Round one: teleconference

Panels members were asked to consider the best mode of data collection from the mothers of the infants from one to 6 months of age. Options discussed included phone call, face-to-face, text message, online diary using phone application or weblink. The panel recommended phone calls or face to face (with use of text message to organise time/venue). The panel advised that women were unlikely to use a mobile phone application to report data. The panel recommended gaining feedback from Indigenous women on their preference for the modality of data collection i.e. phone call, face-to-face, email, mobile phone application. Options discussed for personnel to collect data included an on-site research facilitator (a volunteer for the service who would be aiding the main trial) or other female health worker with a trusted relationship with the woman. The panel members advised additional information would be required to form a decision on the inclusion of respiratory items and requested input from Respiratory Paediatrician (JM) as required to support decision making.

### Round two: questionnaire

Forty-eight items were presented in total for consideration. Five items were presented on how data should be collected (frequency, number of survey questions, modality, personnel to collect data and reimbursement amount) (Table 2). Two items reached consensus, 1) frequency of data to be collect monthly rather than fortnightly and 2) modality of collection for women to choose their preference. Three items progressed to round three (number of survey questions, personnel to collect data, reimbursement amount). Forty-three items were presented on acute respiratory symptoms, health care utilisation, exposure to tobacco smoke and breastfeeding status. Of the 43 items, one item reached consensus (exposure to tobacco smoke). Twenty-eight items progressed to round three and 16 items were omitted (Table 3).

**Table 2:** Frequency, number of questions, mode, personnel to collect data and reimbursement

Items	Round 2 ***n*** = 8	Results 3 ***n*** = 8	Consensus
Frequency of data collection:			
Fortnightly	0	–	
Monthly	7	7	✓
Number of questions:			
1 to 5	0	–	
6 to 10	4	5*	✓
11 to 15	1	1	
16 to 20	2	2	
Modality			
Phone call	3	–	
Survey	2	–	
Phone app	2	–	
Email	1	–	
Women’s preference	7	7	✓
Women randomised to different modality	1	–	
Who should collect data			
Research facilitator (based on site, Indigenous or non-Indigenous)	6	2	
Indigenous researcher (based at research institution)	5	3	
Non Indigenous researcher (based at research institution)	2	–	
Research facilitator, if not possible, Indigenous researcher	–	3*	
Unsure	1	–	✓
Reimbursement to mother, amount per survey:			
$15 voucher	3	1	
Baby bundle (value of $15)	3	2	
v$10 voucher	1	–	
$5 voucher	0	–	
Research site to choose either $15 or $ baby bundle	–	5*	✓

* Rule enacted, highest frequency accepted if consensus not achieved in Round 3

**Table 3:** Consensus for outcomes for acute respiratory symptoms, health care utilisation, and exposure to tobacco and breastfeeding status from 1 to 6 months of age

Item	Round 2 ***n*** = 8	Round 3 ***n*** = 8	Consensus
Has your baby had wheeze or whistle in the past 4 weeks?	4	7	✓
Has your baby had a moist or wet cough in the past 4 weeks?	6	7	✓
Has your baby had a dry cough in the past 4 weeks?	6	7	✓
Has your baby had shortness of breath in the past 4 weeks?	4	7*	✓
Has your baby had an earache in the past 4 weeks?	4	7*	✓
Has your baby had a runny nose in the past 4 weeks?	4	7*	✓
Does your baby have a cough today?	6	5*	✓
Have you been worried about your baby’s health for any reason in the past 4 weeks?	5	7*	✓
If yes, what have you been worried about?	4	8*	✓
Has your baby been hospitalised in the past 4 weeks?	6	7*	✓
If yes, what were the reasons your baby went to hospital?	5	7*	✓
If yes, how many days was your baby hospitalised?	6	7*	✓
Has your baby been to see a doctor at any time in the past 4 weeks?	5	7*	✓
If yes, what were the reasons?	5	7	✓
Has your baby been given medications in the past 4 weeks?	6	7*	✓
Has exposure to tobacco smoke changed?	7	–	✓
Has breastfeeding changed in the past 4 weeks?	6	8*	✓
Any out of pocket expenses to care for your baby’s sickness?	4	3	✘
Has your baby had any feeding difficulties in the past 4 weeks?	4	3	✘
Has your baby had a fever/temp/feel hot in the past 4 weeks?	2	–	✘
Has your baby had chills in the past 4 weeks?	1	–	✘
Has your baby vomited in the past 4 weeks?	1	–	✘
Has your baby had diarrhea in the past 4 weeks?	1	–	✘
Has your baby had irritability in the past 4 weeks?	0	–	✘
Has your baby had increased tiredness in the past 4 weeks?	0	–	✘
Has your baby had unsettled sleep in the past 4 weeks?	0	–	✘
Has your baby had fast breathing in the past 4 weeks?	4	0	✘
How many days has your baby had the cough for?	6	6	✘
Are you worried about your baby’s cough becoming worse?	5	1	✘
What is your baby’s cough like in daytime?	5	0	✘
What is your baby’s cough like in night time?	5	0	✘
Total number of days the baby was in hospital.	3	**–**	✘
Anything else that affects your family getting health care for your baby?	4	3	✘
If yes, how many times has the baby been to the doctor?	3	–	✘
Total number of days baby was in hospital	3	–	✘
Amount of time spent from work/home to get health care for baby?	3	–	✘
How many hours per week have been spent getting health care for your baby?	1	–	✘
Has your baby been given antibiotics in the past 4 weeks?	6	1	✘
What is the name of the hospital?	0	–	✘
Has any person in the baby’s household had a respiratory illness?	2	–	✘
Has your baby seen any other health professional?	5	4	✘
How many times has your baby been to see the health professional?	3	5	✘
Reason (s) baby seen by other health professional	3	7	✘
**Total**	**43**	**28**	**17**

* Rule of combining ‘very essential’ and ‘somewhat essential’ enacted

### Round three: questionnaire

Thirty-one items were presented in total. Of the three items presented on how data should be collected, number of questions was 5 to 10, site to choose personnel to collect data and site to choose $15 gift card or $15 baby bundle. Of the 28 measures to be collected presented in round three, 17 were accepted (see Additional file 2 for final version of monthly survey). Five items reached consensus by achieving a response frequency of ≥80% and 12 items reached consensus through enacting the rule to combine votes for ‘very essential’ and ‘somewhat essential’. Items accepted include seven acute respiratory symptoms, two general health items, six items on health care utilisation, one item on exposure to tobacco smoke and one item on breastfeeding status. Additional recommendations from the panel were to provide families and health providers with education on detecting and managing chronic cough, and to ensure adequate follow up of infants with chronic cough.

#### Measures for respiratory illness and development for 6 months old infants

### *Round One*: teleconference

Five measures were discussed, 1) 50-item parent report respiratory symptom screening questionnaire [30], 2) 18-item respiratory screening questionnaire adapted into Creole [31], 3) a clinical assessment form developed for the purpose of the larger study, 4) Ages and Stages Questionnaire (ASQ) [32] and 5) an adapted version of ASQ for remote Indigenous communities, ASQ-TRAK [33]). Participants were not aware of any other suitable measures or existing surveys.

### Round two: questionnaire

Of the five assessments tools, none reached consensus for use in the existing form. Qualitative feedback from the panel recommended a shorter length questionnaire. The questionnaire adapted into Creole language from the Torres Strait was not considered suitable for most Indigenous women. Participants recommended specific language changes or inclusion of definitions for words such as ‘posset’, ‘wheeze’ and, ‘rattles/ruttles’. Minor feedback was received on the clinical assessment form including a recommendation to ask more broadly about a child’s respiratory health and then use prompts for specific respiratory conditions, e.g. bronchitis.

Five of eight participants indicated it was important to collect developmental outcomes at 6 m and five of eight indicated that the ASQ and ASQ TRAK were suitable tools. Key feedback on how the data should be collected included: a health professional should complete it with the woman and infant, the health professional must be familiar with working in Indigenous communities, and the questionnaire should be completed prior to a clinical assessment and the results provided to the clinical assessor.

#### Feedback from indigenous women

Overall feedback from the Indigenous women indicated a preference for the 50-item questionnaire compared to the 18-item questionnaire adapted into Creole. There was an overwhelming consensus to shorten the length and clarify certain terms, such as ‘posset’ and ‘rattly breathing’. Similar to the Indigenous panel, women advised that the Creole language was only suitable for Indigenous people who speak Torres Strait Creole. Women also recommended a simpler layout, particularly if surveys are to be parent completed.

### Round three

Based on the feedback gathered from participants, several changes were made to the 6 months of age questionnaires presented in round three. The 50-item questionnaire was reduced to 33- items (see Additional file 3). The clinical assessment form was reduced to one page and included growth parameters, immunisations, respiratory illnesses since birth, other significant illness since birth, and current medications. The clinical assessment form (see Additional file 4) was recommended to be completed with information extracted from the clinical notes and parent report. A consensus from participants, 8/8 (100%), was achieved for use of the three assessment tools in their amended form.

## Discussion

A modified Delphi process was completed with eight Indigenous experts, and focus groups were conducted with 18 Indigenous women about culturally safe measures for Infant respiratory health. To our knowledge, this is the first consensus-based study on measures for detecting respiratory illness in Indigenous Australian infants. Measures that reached consensus included 15 measures at birth, 17 measures from 1 to 6 months of age, and three questionnaires to be used at 6 months of age. The preferred mode for data collection differed for the different time points. Consensus was reached that birth measures should to be collected via a hospital discharge summary, 1 to 6 month measures via parent report with mode decided by woman i.e. phone call, mobile phone application, or online survey, and 6 months of age measures collected using parent report questionnaires completed with a trusted health professional in conjunction with clinical notes.

Birth measures had a high rate of inclusion (15/15), which might be due to the standard nature of measures and minimal burden to participating women. In contrast, respiratory symptoms collected on a regular basis were much slower to reach consensus with only five items accepted for inclusion in rounds two and three. The five items were ‘wheeze/whistle’, ‘moist/wet/cough’, ‘dry cough’, ‘reasons for seeing a doctor’ and ‘change in exposure to tobacco smoke’. These are well aligned with the literature. Wheeze is the most reliable symptom to detect asthma [35] and wet cough for bronchiectasis [4, 9, 36]. Seeing a doctor may indicate severity, and exposure to environmental tobacco smoke during infancy doubles the risk of hospitalisation for respiratory illness in infancy [37], so an important variable to collect.

Two potential respiratory questionnaires for use at 6 months of age were presented to the panel It was consistent between the panel and women in the focus groups that Torres Strait Creole is not suitable for most Indigenous women, though a questionnaire with fewer items was preferred. The language of the 50 item questionnaire was largely understood and accepted by women, which is unsurprising as it stems from the ISAAC protocol which has been tested in 97 countries [38]. The 50-item questionnaire was ultimately shortened to 33 items based on feedback. A developmental screening measure, the Ages and Stages questionnaire [39] as well as the adapted version for remote Indigenous communities [33] were also presented to the panel. Interestingly all panel members indicated inclusion of a measure on child development, when not typically measured in studies on respiratory health. The strong interest to include a developmental measure raises the question of what other measures may be important, and perhaps more meaningful to Indigenous communities. Other less commonly reported measures in child respiratory studies include child parent quality of life [40, 41] and child functioning [42].

This study had several limitations. The involvement of Indigenous women was limited. Women participated in one focus group to provide feedback on one type of measure (6 months of age respiratory questionnaires); we did not obtain final feedback from women on changes made to the questionnaire recommended by the expert panel (removal of 17 items). The measures identified in this study may be more confidently used if greater end user involvement had occurred [43]. While we strongly acknowledge the importance of end-user involvement, the focus here was to gain expert consensus from Indigenous academics and clinicians on essential respiratory measures, future studies should place emphasis on pre-testing the identified measures with end-users from a range of communities. A second limitation was that findings may not be generalisable to the diversity of Indigenous peoples of Australia. While panel members were from different regional, remote and urban communities, the number of panel members was relatively small and women were from NSW communities only. The number of participants in a Delphi study is usually 11 to 25, though less than 10 is also common [44]. A third and important limitation was that the measures identified focus on a rather short period in a child’s life, birth to 6 months of age. The 6 months age range was of focus as it is the follow-up period of the larger trial. As many chronic respiratory illnesses only develop later in childhood and are uncertain in infancy, e.g. asthma and bronchiectasis, accepted measures for use throughout childhood are needed. Lastly, if further rounds of consensus were completed the number of items may have been reduced, which can result in higher response rates for trials [45]. An important consideration to be examined if pre-testing of measures.

The strength of this study was the engagement of Indigenous experts from several disciplines to work together and identify a comprehensive set of respiratory measures in the context of cultural safety for Indigenous infants. Knowledge was generated with Indigenous academics, clinicians and women to optimise the cultural safety of data collection in a trial examining infant respiratory outcomes. The measures identified are for a number of time points in the first 6 months of life using a range of sources (medical records, parent report and observation). A range of sources is important given the known pitfalls of relying on any one of these sources alone [14].

A modified Delphi process may be a useful method to systematically involve Indigenous people in decisions for trials. The Delphi has been used in other areas of Indigenous health research including to develop mental health guidelines [20] and data collection strategies for maternity experiences [21]. Other high-level consultative methods to develop measures for use with Indigenous people have also been used. A recent example is the development of a survey for the Mayi Kuwayu Study, a national longitudinal study on adult Indigenous Australian well-being [46]. Consultation was completed with 165 Indigenous peoples attending 24 focus groups across Australia from 2014 to 2017. Pilot testing of the survey was completed with 160 and 209 Indigenous participants. A second example is the Healing the Past by Nurturing the Future study, a study in part to develop a measure to identify complex trauma experienced by Indigenous parents [47]. Consultation includes four large-scale co-design workshops across three States with Indigenous parents, service providers, community leaders, researchers and wider community members. Comprehensive consultation is expected from conception to conclusion in research with Indigenous peoples [48]. With varying methods and approaches for consultation, a Delphi methodology is one approach that can provide a systematic, transparent and feasible process for expert consensus in trials.

The Indigenous panel that participated in the consensus process made two important unexpected recommendations that may aid more accurate data collection and increase recruitment and retention in trials. The first was to provide education to participating families and health providers on respiratory symptoms and management pathways. This recommendation aligns with a recent qualitative study with 40 Indigenous community members reporting 70% considered chronic cough normal in children [49]. By providing culturally appropriate definitions on respiratory terms such as wheeze and wet cough, and information on the importance of seeking treatment, the accuracy of parent report may improve and lead to better disease detection and optimal treatment [50]. The second recommendation was to provide adequate follow up of participating infants. Cough guidelines recommend children aged 14 years or less with a chronic cough of 4 weeks should have a chest radiograph and spirometry test (when age appropriate) [51]. In research studies on infant respiratory health, we have opportunity and ethical responsibility [48] to ensure that children receive adequate treatment during and on study completion. Studies designed with a reciprocal approach including assured access to quality treatment may improve retention rates, as in a recent study on incidence of respiratory illness in Queensland [11].

This is a preliminary step in developing a set of standard measures to detect respiratory illness in community based Indigenous infants. Future research is needed to test the validity of the identified measures for use in trials and practice. The 6 month respiratory questionnaire has been found to have good repeatability, though the authors acknowledge that validity testing is needed [30]. We anticipate that results from the larger trial will allow for comparison of self-report to clinical notes which will give indication of validity for certain questions including questions on health service utilisation and diagnosed respiratory illness. To validate questions on acute respiratory symptoms such as runny nose, ear ache, wheeze, shortness of breath, and cough, a comparison to objective measures such as recordings of cough or wheeze, and clinical observation is needed [30]. This is a resource intensive process that may involve twice weekly home visits [52] or potentially video conferencing. While it was not feasible for the measures to be validated as part of this study, the process we undertook in it’s development consulting with a range of consumers and stakeholders has contributed to strengthening the tools face validity when used with Indigenous Australians. Additional considerations for testing these measures may include information for families to combat the normalisation of respiratory illness [53], flexible mode of delivery given the many other needs and problems Indigenous families experience [19], and trusted and skilled interviewers to ensure cultural safety.

## Conclusions

A modified Delphi process with Indigenous multi-disciplinary experts determined culturally safe measures to identify respiratory illness in Indigenous infants from birth to 6 m of age. We set out to develop a set of measures that would meet the needs of families, clinicians and researchers that were culturally safe and feasible. In total, 15 items for birth, 17 items from 1 to 6 months and 3 surveys for use at 6 months of age were identified. Future studies are required to assess the validity and reliability of and participation in surveys using these relevant and acceptable measures.

## Supplementary information

**Additional file 1.** Birth outcomes data extraction form.

**Additional file 2.** Acute respiratory symptoms, health care utilisation, and environment monthly survey.

**Additional file 3.** Respiratory questionnaire for infants (6 months).

**Additional file 4.** Six-month clinical assessment form.

## Data Availability

The datasets supporting the conclusions of this article are included within the article and its additional files. Additional files include: Additional file 1 Birth outcomes data extraction form Additional file 2 Acute respiratory symptoms, health care utilisation, and environment monthly survey Additional file 3 Respiratory questionnaire for infants (6 months) Additional file 4 Six-month clinical assessment form

## Abbreviations

IAHA: Indigenous Allied Health Australia
Australian: Indigenous HealthInfoNet
CATSINaM: The Congress of Aboriginal and Torres Strait Islander Nurses and Midwives
NICU: Neonatal Intensive Care Unit
ASQ: Ages and Stages Questionnaire
