# Supplementary material for: Respiratory, birth and health economic measures for use with Indigenous Australian infants in a research trial: a modified Delphi with an Indigenous panel
Source: BMC Pediatr. 2020 Aug 5;20:368. doi: 10.1186/s12887-020-02255-x (PMC7409441; doi:10.1186/s12887-020-02255-x)
Supplement: Supplementary file 1 — Additional file 1. Birth outcomes data extraction form. [file 12887_2020_2255_MOESM1_ESM.docx]

**Additional file 1**

**Birth outcomes data extraction form**

| **Participant details and birth outcomes** | |
| --- | --- |
| Health of mother |  |
| Number or people living in home |  |
| Education attainment of mother |  |
| Place of residence |  |
| Labour (induction/spontaneous) |  |
| Birth type (caesarean, vaginal) |  |
| Substance use in pregnancy |  |
| Gestational age |  |
| Weight (kg) (%) |  |
| Length (cm) (%) |  |
| Head circumference (cm) (%) |  |
| Sex |  |
| Apgar score |  |
| Cord blood gas values |  |
| NICU admissions |  |

**Note:** To be extracted from discharge summary and clinical notes.
