# Supplementary material for: Respiratory, birth and health economic measures for use with Indigenous Australian infants in a research trial: a modified Delphi with an Indigenous panel
Source: BMC Pediatr. 2020 Aug 5;20:368. doi: 10.1186/s12887-020-02255-x (PMC7409441; doi:10.1186/s12887-020-02255-x)
Supplement: Supplementary file 2 — Additional file 2. Acute respiratory symptoms, health care utilisation, and environment monthly survey. [file 12887_2020_2255_MOESM2_ESM.docx]

| **Additional file 2**  **Acute respiratory symptoms, health care utilisation & environment infant survey (2,3,4,5,6 month)** |
| --- |
| 1. Has your baby had any of these symptoms in the past 4 weeks (select any that apply)? 2. Runny nose Yes No 3. Earache/ear discharge Yes No 4. Wheeze/whistle Yes No 5. Shortness of breath Yes No 6. Moist/wet/gurgly cough Yes No 7. Dry cough Yes No   If yes (to cough e or f ):  **1a:** Does your baby have the cough today? Yes No  (If yes to “does your baby have a cough today?”  Note to interviewer: send question weekly (day 7, 14, 21, 28) until mother responds “no”) |
| 1. Have you been worried about your baby's health for any reason in the past 4 weeks? Yes No   If yes:  **2a.** What have you been worried about? [open text] |
| 1. Has your baby been hospitalised for any reason in the past 4 weeks?  Yes No   **3a:** How many days was the baby in hospital for (including multiple admissions)? [Open text]  **3b:** What were the reasons the baby went to hospital (tick any that apply)?  Lower respiratory tract infection (e.g. bronchiolitis, cough, breathing problems, chest infection)  Upper respiratory tract infection (e.g. ear infection, sore throat, ‘cold’)  Gastrointestinal infection (e.g. vomiting or diarrhoea)  Other (open text) |
| 1. Has your baby been to see a doctor, nurse or any other health professional in the past 4 weeks (not including hospital admissions)? Yes No   If yes:  **4a**: What were the reasons the baby saw the health professional?  Vaccination/ routine baby health check  Lower respiratory tract infection  Upper respiratory tract infection  Gastrointestinal infection  Other (open text) |
| 1. Has your baby been given any medications in the past 4 weeks? Yes No |
| 1. Has the baby's exposure to tobacco smoke changed in the last month? Yes No   **6a**: If yes,  No household exposure: household members stopped smoking inside  Less household exposure: household members have reduced smoking inside  More household exposure: (there is more smoking inside) |
| 1. If your baby was being breastfed at the time of the last survey, has breastfeeding type changed?   Yes, gone from fully breastfed to partially breastfed  Yes, stopped breastfeeding completely  No change  Not applicable, baby not breastfed when I completed last survey  Unknown |
