# Supplementary material for: Respiratory, birth and health economic measures for use with Indigenous Australian infants in a research trial: a modified Delphi with an Indigenous panel
Source: BMC Pediatr. 2020 Aug 5;20:368. doi: 10.1186/s12887-020-02255-x (PMC7409441; doi:10.1186/s12887-020-02255-x)
Supplement: Supplementary file 3 — Additional file 3. Respiratory questionnaire for infants (6 months). [file 12887_2020_2255_MOESM3_ESM.doc]

**Additional file 3**

**Respiratory Questionnaire for infants (6 months)**

***Questions on wheezing***

By “wheezing” we mean breathing that makes a high-pitched whistling or squeaking sound from the chest, not the throat.

1. **Has your child ever had wheezing or whistling in the chest at any time in the past?**

Yes 🞎 No 🞎

• If you answered “no” please skip to question 10

1. **How old was your child when he/she first began to wheeze?** Months __________________
2. **In the last 6 months, has your child had wheezing or whistling in the chest during or soon after a cold or flu?**

Yes 🞎 No 🞎

1. **In the last 6 months, has your child had wheezing or whistling in the chest even without having a cold or flu?**

Yes 🞎 No 🞎

1. **How many attacks of wheezing has your child had during the last 6 months?**

None 🞎 1 to 3 🞎 4 to 12 🞎 More than 12 🞎

1. **Do these attacks cause him/her to be short of breath?**

Yes, always 🞎 Yes, occasionally 🞎 No, never 🞎

1. **Which of these two descriptions fits best your child’s wheeze? (tick one only)**

My child has only short attacks of wheeze, for example with colds.

In between these attacks, he/she does not normally wheeze 🞎

My child wheezes always or a lot of the time. With colds he/she

has attacks with more severe wheeze 🞎

1. **In the last 6 months, how often, on average, has your child’s sleep been disturbed due to wheezing?**

Never woken with wheezing 🞎

Less than one night per week 🞎

One or more nights per week 🞎

1. **In the last 6 months, how much did wheezing interfere with your child’s daily activities?**

Not at all 🞎

A little 🞎

A moderate amount 🞎

A lot 🞎

1. **Looking back on the last 6 months, do you think that your child had asthma?**

Yes 🞎 No 🞎

1. **In the last 6 months, did your child suffer from rattly breathing (ruttles)?**

Never 🞎

Only with a cold 🞎

Sometimes 🞎

Even without a cold 🞎

Almost always 🞎

1. **Does your child attend day care, nursery school or play school?**

Yes 🞎 No 🞎

1. **Was your child breastfed?**

Yes 🞎 No 🞎

•If yes, how long:

Less than a month 🞎

1-3 months 🞎

4-6 months 🞎

More than 6 months 🞎

1. **During the first 6 months of life, did your child vomit?**

Not at all 🞎 A little 🞎 A lot 🞎

1. **Has your child ever suffered from any of the following conditions?**

• Pneumonia? Never 🞎 Once 🞎 More than once 🞎

• Whooping cough? Never 🞎 Once 🞎 More than once 🞎

• Bronchiolitis? Never 🞎 Once 🞎 More than once 🞎

• Croup? Never 🞎 Once 🞎 More than once 🞎

1. **Has your child had this itchy rash at any time in the last 6 months?**

Yes 🞎 No 🞎

1. **In the past 6 months, has your child had eczema?**

Yes 🞎 No 🞎

***Questions on ears, nose and throat***

1. **In the last 6 months, how many times has your child had a cold or flu?**

Never 🞎

1 - 3 times 🞎

4 - 6 times 🞎

7 -10 times 🞎

More than 10 times 🞎

1. **How long does a cold usually last in your child?**

Less than 1 week 🞎

1 to 2 weeks 🞎

2 to 4 weeks 🞎

More than 4 weeks 🞎

1. **In the past 6 months, has your child had a problem with sneezing, or a runny, or blocked nose when he/she did NOT have a cold or the flu?**

Yes 🞎 No 🞎

1. **Over the past 6 months, has your child snored at night?**

Yes 🞎 No 🞎

• If yes, how often:

Only with a cold 🞎

Sometimes even without a cold 🞎

Almost always 🞎

1. **In the past 6 months, has your child had ear infections?**

No, never 🞎

Yes, once 🞎

Yes, more than once 🞎

***Questions on coughing***

1. **Does your child usually have a cough with colds?**

Yes 🞎 No 🞎

1. **Does your child have a cough even without having a cold?**

No, never 🞎 Yes, sometimes 🞎 Yes, always 🞎

1. **In the last 6 months, has your child had a dry cough at night, apart from a cough associated with a cold or a chest infection?**

Yes 🞎 No 🞎

***Questions on the household and family***

1. **Does your child have brothers and sisters?**

Yes 🞎 No 🞎

- How many? ____ (please fill in number)
- How many of them have:
- Asthma or wheezing? ____ (please fill in number)
- Hayfever? ____ (please fill in number)
- Eczema? ____ (please fill in number)

1. **Has the child’s father ever suffered from any of the following conditions?**

• Asthma or wheezing? Yes 🞎 No 🞎 Don’t know 🞎

• Bronchitis? Yes 🞎 No 🞎 Don’t know 🞎

• Hayfever? Yes 🞎 No 🞎 Don’t know 🞎

• Eczema? Yes 🞎 No 🞎 Don’t know 🞎

1. **Has the child’s mother ever suffered from any of the following conditions?**

• Asthma or wheezing? Yes 🞎 No 🞎 Don’t know 🞎

• Bronchitis? Yes 🞎 No 🞎 Don’t know 🞎

• Hayfever? Yes 🞎 No 🞎 Don’t know 🞎

• Eczema? Yes 🞎 No 🞎 Don’t know 🞎

1. **Which fuel is mainly used for cooking in the home?**

Electricity 🞎

Gas 🞎

Other fuel 🞎

1. **How do you heat your home? (tick as many as apply)**

Central heating 🞎

Gas heaters in rooms 🞎

Coal or wood fire 🞎

Other (electricity, oil) 🞎

1. **Do you keep any household pets?**

Yes 🞎 No 🞎

• If yes, do you keep any of these pets? (tick as many as apply)

Dog 🞎 Cat 🞎 Other furry pets 🞎 Bird 🞎

1. **How would you describe the location of your house?**

In a street with very dense traffic (main road) 🞎

In a street with moderate traffic (residential road) 🞎

In a quiet street with little or no traffic 🞎

1. **Did you have problems understanding this questionnaire?**

Yes 🞎 No 🞎

Please write any comments you have about your child’s health or about the questionnaire in the space below:
