# Supplementary material for: Respiratory, birth and health economic measures for use with Indigenous Australian infants in a research trial: a modified Delphi with an Indigenous panel
Source: BMC Pediatr. 2020 Aug 5;20:368. doi: 10.1186/s12887-020-02255-x (PMC7409441; doi:10.1186/s12887-020-02255-x)
Supplement: Supplementary file 4 — Additional file 4. Six-month clinical assessment form. [file 12887_2020_2255_MOESM4_ESM.docx]

**Additional file 4**

**6 Month Clinical Assessment Form**

| **Participant details** | | | |
| --- | --- | --- | --- |
| Subject ID (child) |  | Date |  |
| MAP ID (mother) |  | Infant’s age (months) |  |
| **Growth parameters** | | | |
| Weight (kg) (%) |  | Head circumference (cm) (%) |  |
| Length (cm) (%) |  |  |  |
| **Immunisations** | | | |
| Immunisations (2/12) |  | Immunisations (4/12) |  |
| Immunisations (6/12) |  |  |  |
| **Respiratory Illnesses since birth** (including pneumonia, bronchiolitis, protracted bacterial bronchitis-CSLD, upper respiratory tract conditions, otitis media/effusion) | | | |
|  | | | |
|  | | | |
|  | | | |
| **Other significant illnesses/surgery since birth :** | | | |
|  | | | |
|  | | | |
|  | | | |
|  | | | |
| **Current Medications prescribed** (include dose, name of medication, number and timing of doses) (medications may include puffer, steroids, vitamins, antibiotics, traditional medicine or others): | | | |
|  | | | |
|  | | | |
|  | | | |
|  | | | |

**Note:** This clinical assessment is to be completed by a health professional (e.g. nurse, GP or paediatrician) with family member and baby at 6 months (+/- 1 month).It is preferable that the developmental assessment (Ages and stages questionnaire) is completed prior to this clinical assessment and results are provided to health professional prior this assessment (if clinician is different).
